# Toxic Effects of Two Representative Rare Earth Elements (La and Gd) on *Danio rerio* Based on Transcriptome Analysis

**DOI:** 10.3390/toxics10090519

**Published:** 2022-08-31

**Authors:** Shu Kang, Cheng Guo, Chenyang Xue, Chenshu Ma, Huaizhong Mu, Lizong Sun

**Affiliations:** 1 School of Public Management, Liaoning University, Shenyang 110016, China; 2 Key Laboratory of Pollution Ecology and Environmental Engineering, Institute of Applied Ecology, Chinese Academy of Sciences, Shenyang 110016, China; 3 School of Environmental and Safety Engineering, Liaoning Petrochemical University, Fushun 113001, China; 4 Liaoning Economic Vocational Technological Institute, Shenyang 110016, China

**Keywords:** rare earth elements, toxicity pattern, zebrafish, homogeneity, transcriptome

## Abstract

The expanding applications of rare earth elements (REEs) in various fields have raised concerns about their biosafety. However, previous studies are insufficient to elucidate their toxic effects and mechanisms of action and whether there are uniform or predictable toxicity patterns among REEs. Herein, we investigated the toxic effects of two representative REEs (lanthanum (La) and gadolinium (Gd)) on zebrafish (*Danio rerio*) through toxicity experiments and transcriptome analysis. The results of the toxicity experiments showed that the two REEs have similar lethality, with half-lethal concentrations (LC50) at micromolar levels and mixed toxicity showing additive effects. Differential expression gene screening and functional group enrichment analysis showed that La and Gd might affect the growth and development of *Danio rerio* by interfering with some biological molecules. The two REEs showed significant effects on the metabolic pathways of exogenous or endogenous substances, including glutathione sulfotransferase and acetaldehyde dehydrogenase. Moreover, some basic biological processes, such as DNA replication, the insulin signaling pathway, and the p53 signaling pathway, were significantly enriched. Overall, the toxicity patterns of La and Gd may affect some biological processes with different intensities; however, there are many similarities in their toxicity mechanisms and modes of action. The concentrations investigated in this study were comparable to those of REE residues at highly contaminated sites, thus mimicking the ecotoxicological effects at environmentally relevant concentrations.

## 1. Introduction

Rare earth elements (REEs) include 15 lanthanides (Ln) (ranging from lanthanum (Z = 57) to lutetium (Z = 71)), as well as scandium and yttrium. Owing to their irreplaceable material properties, REEs have become an important strategic resource and are widely used in the fields of electronics, metallurgy, energy, medicine, and agriculture [1,2]. In the past few decades, the global demand for REEs has increased exponentially, in turn disrupting their biogeochemical cycle [3,4]. Moreover, the high demand for REEs has triggered rapid growth in the mining industry. Nonetheless, REEs readily deposit into the environment and are, thus, regarded as emerging contaminants [5,6,7]. Recent studies have shown that REEs are abundantly enriched in soils and plants in mining areas [8]. Their concentrations in topsoil are more than 10 times the background value and are also increasingly deposited in aquatic ecosystems [9]. Owing to this, their biosafety and related ecotoxicological effects are receiving increasing attention [10,11,12,13,14,15,16].

Compared to extensive studies that have been performed on heavy metals (Cu, Zn, Cd, and Pb), only a few studies have reported on the ecotoxicity of REEs [9]. Most existing studies on REEs have focused on geological, mineralogical, and material properties, with their environmental risks receiving little attention [17]. Presently, the reported toxicity data for REEs are mostly concentrated on single elements, especially lanthanum (La) and cerium (Ce) [18,19,20]. To predict and explain the biological effects of other REEs, it is important to understand and determine whether extrapolation can be applied to deduce the properties of other REEs based on the ecotoxicity of one REE. Gonzalez et al. [21] statistically analyzed hundreds of reports on the toxicity of REEs in aquatic organisms and found that the toxicity data in literature immensely varied, failing to determine any form of regular pattern or correlation that could be inferred from similarities in their atomic properties.

To date, few studies have reported on the mixed toxic effects of REEs on aquatic organisms [22,23,24,25,26,27]. Tai et al. [22] found that the biological toxicities of 13 REEs to unicellular algae (*Chlorella autotrophica*) were essentially the same, and the toxicity of the mixture of components was similar to that of a single element. Blinova et al. [23] found that the lethal concentrations (LC50) of five REEs in freshwater crustaceans were between 0.3 and 0.5 mg L^−1^, suggesting that REEs can be regarded as a uniform group of elements with an additive toxicity model despite their mechanisms of action revealed. However, Romero-Freire et al. [24,25] found that REEs are more toxic to low-trophic organisms, with mixed toxicity having an additive effect on low-trophic organisms (such as bacteria) and an antagonistic effect on high-trophic organisms (such as zebrafish). Bing et al. [26] found that three REEs (Y, La, and Ce) had similar K_m_ and J_max_ values through comparison using a simple biological ligand model. However, Yang et al. [27] concluded that the ligand model could not adequately explain the toxicity of REEs because the mixtures of different components are unlikely to have exactly the same or independent modes of action. Thus, it is difficult to compare the general patterns of REEs in terms of toxicity data from the currently available literature. Owing to this, a comparative study on the toxicity of REEs should not rely exclusively on model predictions and toxicity data. Along with paying attention to exposure conditions, tested species, and test endpoints, it is also necessary to clarify the toxicity mechanism of REEs at the molecular level to determine whether it shows a predictable model of the toxicity effects and mechanisms [21,26,27].

Transcriptome analysis has been efficiently applied in risk assessments of chemical substances and ecotoxicological mechanism investigations [28]. Compared to traditional toxicological analysis, transcriptome analysis has obvious advantages in distinguishing toxicity differences and action patterns of harmful substances [29,30]. Therefore, in this study, two representative REEs, La and gadolinium (Gd), were selected as test elements to explore their ecotoxic effects and mechanisms in zebrafish (*Danio rerio*). This study aimed: (1) to assess the toxic effects and modes of action of the two REEs and their mixtures on *Danio rerio*, (2) to clarify their toxic mechanisms based on transcriptome analysis, and (3) to investigate the toxicological differences and potential toxicity patterns between the two REEs.

## 2. Materials and Methods

### 2.1. Materials and Reagents

Zebrafish (*Danio rerio*, AB strain), about 2 months old with body lengths of 2.0 ± 0.5 cm were purchased from the Wuhan Institute of Aquatic Biology, Chinese Academy of Sciences. The fish were raised in an aquatic system. They were housed under the following conditions: a light/dark cycle of 14/l0 h, water temperature maintained at 26 ± 1 °C, and a pH of 7.0 ± 0.5. After domestication in the experimental environment for 2 weeks, no obvious diseases or deformities were visible. The selected two representative REEs (LaCl_3_6H_2_O and GdCl_3_6H_2_O (purity > 99.9%)) were purchased from Sterem Chemicals (MA, USA). These two REEs, which are relatively rich in the earth’s crust, are representative of odd REEs (atomic number: 57La) and even REEs (64Gd) and are representative of light and heavy REEs, respectively.

### 2.2. Acute Toxicity Test

The culture and acute toxicity tests of *Danio rerio* were performed as per the protocol outlined in the fish acute toxicity test (OECD 203) and method for determination of acute toxicity of water quality substances to freshwater fish (zebrafish) (GB/T13267-91). Standard dilution water was used after aeration for 48 h. For the configuration method, 11.76 g calcium chloride (CaCl_2_·2H_2_O) was dissolved in water and diluted to 1 L, 4.93 g magnesium sulfate (MgSO_4_·7H_2_O) was dissolved in water and diluted to 1 L, 2.59 g sodium bicarbonate (NaHCO_3_) was dissolved in water and diluted to 1 L, and 0.23 g potassium chloride (KCl) was dissolved in water and diluted to 1 L. Then, 25 mL of each of the above four solutions was taken, mixed, and diluted to 1 L with distilled water and aerated until the dissolved oxygen concentration reached the air saturation value. The newly configured standard dilution water had a pH of 7.8 ± 0.2 and a hardness of 250 mg L^−1^. The molarity of each ion in the standard water is shown in Table S1.

Based on the pre-experiment results, a static exposure toxicity test was used to set the concentrations of REEs to 0, 20, 40, 80, 160, and 320 μmol L^−1^, with three replicates being performed for each treatment. Eight zebrafish were placed in each tank (20 × 20 × 25 cm, 10 L) with a water volume of 2 L. Before the beginning of the toxicity test, the 24 h-LC50 of K_2_Cr_2_O_7_ in zebrafish was determined to be 298.7 ± 23.7 mg L^−1^, confirming that the zebrafish were not in distress. In order to ensure that the pH of all treatments was maintained at 7.0, 1 mol L^−1^ NaOH and HCL were used to regulate pH before the toxicity test. Before the test, zebrafish were domesticated in continuously aerated water for more than 7 days under environmental conditions similar to those of the test in terms of water quality and lighting. Feeding was stopped 24 h before the test. The Ln concentrations in the test solution were measured at the beginning and end of the test to detect that the chemical properties of Ln were stable. Dissolved oxygen (DO) concentration, pH, and temperature were measured in each vessel at the beginning and end of the test and once a day. The exposure water was changed every 24 h. Ammonia concentrations were monitored at the beginning and end of each water change. Ammonia concentration measurements during 72 h acute toxicity are shown in Table S2. During this period, fish activity and poisoning symptoms were observed, and dead fish were removed immediately. The number of dead animals was recorded after 72 h. The semilethal concentrations (72 h-LC50) following each treatment were calculated according to the probability unit-logarithmic graphic method.

### 2.3. Bioaccumulation

Based on Section 2.2, three treatments were set up: 0 (control), 0.1 LC50 (low-concentration group, 15 μmol L^−1^), and 0.2 LC50 (high-concentration group, 30 μmol L^−1^). Three replicates were set up for each treatment, and 50 zebrafish were placed in each tank. According to the fish prolonged toxicity test: 14-day study (OECD 204) guidelines, a semistatic test was used for the 28 d toxicity experiment. The test medium was changed once every 2 days, which is fully satisfactory for the chemically stable Ln that the concentration of the test solution fluctuates within 20% of the theoretical concentration value during the change in the solution. The DO concentration, pH, and temperature of each vessel solution were measured twice a week. The Ln concentration in the test solution was measured at the beginning of the test, before the first update of the test solution, and at the end of the test to check that the Ln remained relatively stable during the time of changing the test solution. Feeding was performed once a day, and the feeding amount did not exceed the food requirement of the test fish at one time. The activity of the animals and symptoms of poisoning were observed, and waste and dead fish were cleaned up immediately. At the beginning and the end of the test, the body weight and body length of all surviving zebrafish were measured.

Samples were taken after 28 days of exposure, and muscle, liver, and gills of zebrafish were separated with a scalpel and refrigerated at −80 °C. The Ln content in each treatment was determined by microwave digestion and inductively coupled plasma mass spectrometry (ICP-MS, NexION 300X, Waltham, MA, USA). The pretreatment methods for the determination of Ln in fish tissue and solution were as follows: the former weighed 0.1 g of zebrafish liver tissue dried by gauze (accurate to 0.001 g), and 20 mL of the solution and 5 mL of HNO_3_ were added to the autoclave. The lid was screwed tightly, left for 1 h, and the standard operation procedure of microwave digestion apparatus (APL Touchwin 2.0, Wuhan, China) was followed. After cooling, the lid was slowly opened to vent the air, and the autoclave was placed on a temperature-controlled hot plate to drive out the acid at 140 °C. The digestion solution was transferred to a 10–25 mL volumetric flask, washed three times with a small amount of water, combined in a volumetric flask, and fixed to the scale. The Ln content was determined by ICP-MS. The method was carried out according to the instruction manual of the instrument. The microwave digestion method was based on a previous study [19].

### 2.4. Toxicity of Mixture

The toxic effect of the mixture (Mix) containing La and Gd was determined from an equimolar concentration combination (Mix = ½ La + ½ Gd, Mix = ¼ La + ¾ Gd, and Mix = ¾ La + ¼ Gd). The concentration and toxicity test methods are described in Section 2.2 and Section 2.3. The 72 h-LC50 of zebrafish in each treatment group and the accumulation of La, Gd, and the Mix were measured at the end of the test.

### 2.5. Transcriptome High-Throughput Sequencing

Transcriptome analysis was performed on the livers of the zebrafish collected, as described in Section 2.3. We used the rapid freezing method of 2–4 °C proposed by the American Veterinary Medical Association (2013) for zebrafish euthanized. Liver tissue (100 mg) was ground in liquid nitrogen, and total RNA was extracted using an RNA extraction kit (Takara, Bao Biological Engineering Co., Ltd., Dalian, China). After testing the purity and integrity of the extracted RNA, it was reverse transcribed using the Prime Script TM 1st Strand cDNA Synthesis Kit (Takara) to synthesize cDNA. Specific procedures were performed according to the manufacturer’s instructions.

Based on second-generation sequencing technology, the cDNA library was sequenced using the Illumina high-throughput sequencing platform and transformed by the sequencing platform’s own software to generate raw data for FASTQ. Subsequently, the raw data were filtered to remove low-quality and adaptor sequences to obtain clean data, and sequence comparison was performed with the specified zebrafish reference genome to obtain the comparison data. This process was performed at Shanghai Personalbio Technology Co., Ltd. (Shanghai, China).

Differential gene expression analysis was performed using DESeq to identify differentially expressed genes (DEGs) in zebrafish livers after different treatments. The DEGs were entered in the Gene Ontology (GO) and Kyoto Encyclopedia of Genes and Genomes (KEGG) databases using BLAST software, and their annotation information was obtained.

### 2.6. Quantitative Real-Time Polymerase Chain Reaction

Quantitative real-time polymerase chain reaction (qPCR) was performed using an SYBR Green Mix (20 µL reaction system) amplification system with the following inputs: SYBR (buffer) 10 µL, RF (primer) 2 µL (20 ng), cDNA template (1 µL), and sterile water (7 µL). The PCR amplification process was as follows: predenaturation at 95 °C for 30 s, denaturation at 95 °C for 5 s, annealing at 60 °C for 30 s, annealing at 75 °C for 5 s, 40 cycles, extending at 60 ℃ for 10 min. Fluorescence was measured at the end of the reaction, and real-time amplification and lysis curves were established with three replicates for each treatment. Twelve DEGs (including *CDKA;1*, *CDK2*, *GSTP2*, *UGT5a1*, *ALDH3B1*, *PCNA1*, *CYP1A*, *CDC45*, *TP53*, *BRCA1*, *MRE11*, and *MDM2*) in zebrafish liver were screened using transcriptome high-throughput sequencing. Gene *ACT2* (accession number: 821411) was as an internal reference gene, and the DEGs induced by La and Gd were further verified by qPCR analysis. The genes’ names and their primer sequences are listed in Table S3. The Quant Studio™ Design analysis software automatically calculates the relative expression levels of genes using algorithms based on the 2^−ΔΔCt^ method.

### 2.7. Quality Control and Data Processing

Three biological replicates were performed for all experimental treatments. A multiparameter water quality analyzer (YSI Professional Plus) was used to ensure the stability of the water quality conditions, including pH, temperature, and dissolved oxygen, during the toxicity test. Tukey’s HSD of one-way analysis of variance (ANOVA) was used to analyze significant differences in the control and La- and Gd-exposed bioaccumulation and qRT-PCR validation of DEGs. A value of *p* < 0.05 was considered to indicate a significant difference. Results are given as mean ± standard deviation (SD). The plots were created using Microsoft Excel 2017 and Origin 2017.

## 3. Results

### 3.1. Toxic Effects and Associated Mechanisms of La and Gd on Danio rerio

**Figure 1:**
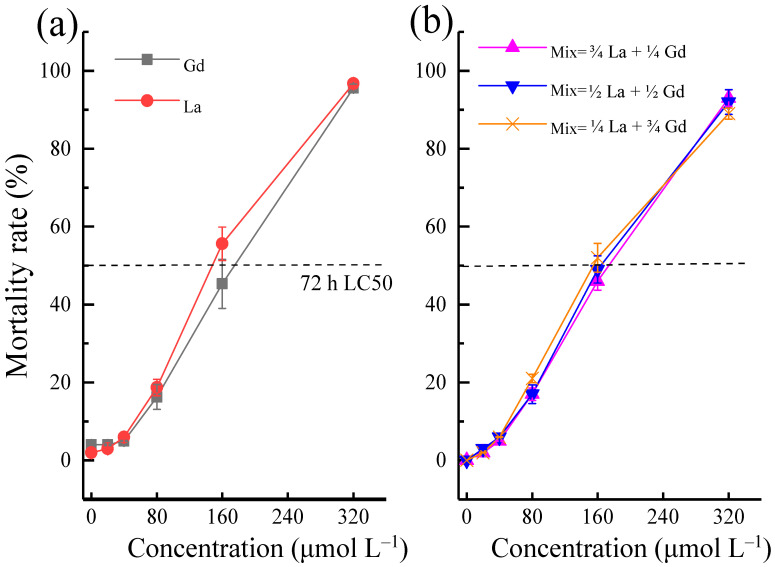
Toxic effects of La, Gd, and the Mix on *Danio rerio*: (**a**) acute toxicity (72 h-LC50) of La and Gd on *Danio rerio*; (**b**) acute toxicity of the mixture (Mix) containing La and Gd determined from an equimolar concentration combination.

Table S4 shows that the variation range of Ln concentration was less than 10%, the DO concentration was not less than 80% of the air saturation value (ASV), the temperature did not vary more than 1 °C, and the pH did not vary more than 0.2 during the test. The acute toxicity test showed that the 72 h-LC50 of La and Gd on *Danio rerio* were 170.73 (161.55–179.56) μmol L^−1^ and 148.86 (141.96–156.32) μmol L^−1^, respectively (Figure 1a). Stressed by the high concentrations of REEs, the death of *Danio rerio* was significantly higher than that observed in the low-concentration and control groups, and the individual action was obviously slow. Compared with La and Gd, the toxicity of the latter on *Danio rerio* was slightly higher than that of the former. As shown in Figure 1b, the 72 h-LC50 of the Mix on *Danio rerio* was 158.57 (149.96–166.73) μmol L^−1^, which is similar to that of a single element, indicating that the Mix had an additive toxic mode.

The survival rate of *Danio rerio* in each treatment group was greater than 95% when they were stressed with 15 μmol L^−1^ and 30 μmol L^−1^ La and Gd for 28 days. The enrichment amounts and bioconcentration factors (*BCF*) of La and Gd in liver, gills, and muscle tissues are shown in Table 1: liver (7.73–9.33) > gills (3.23–4.49) > muscle (0.17–0.33). These findings indicate that REEs were mainly enriched in the livers of *Danio rerio*, which also provided the basis for subsequent transcriptome experiments.

### 3.2. Transcriptome Analysis of Danio rerio under La and Gd Stress

**Figure 2:**
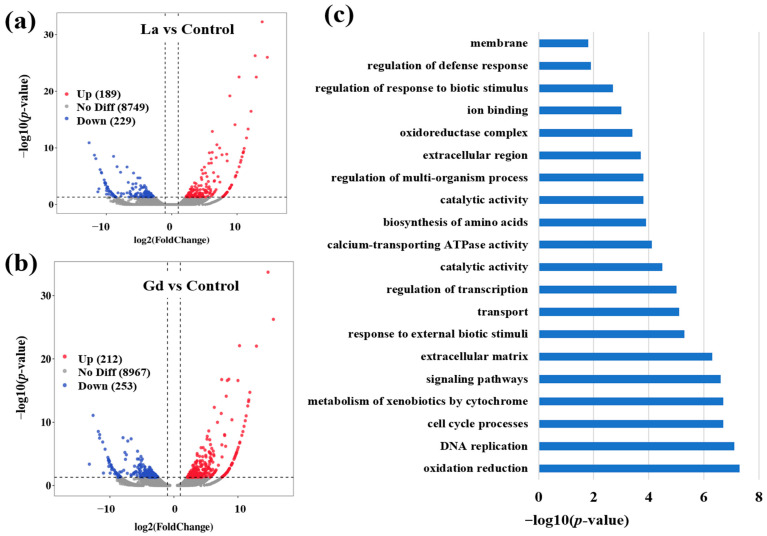
Transcriptome high-throughput sequencing analysis in liver tissues of *Danio rerio* exposed to 30 μmol L^−1^ La and Gd: (**a**,**b**) volcano plots of DEGs in La and Gd, compared with the control, respectively. The red dots indicate upregulated genes, blue dots indicate downregulated genes, and gray dots indicate nonsignificant DEGs; (**c**) analysis of the shared GO classification of DEGs between La and Gd.

Figure 2 and Table S5 show the transcriptome high-throughput sequencing of *Danio rerio* stressed by La and Gd. Compared with the control (Figure 2a), La (30 μmol L^−1^) induced 418 DEGs, of which 189 were upregulated and 229 were downregulated, and Gd (30 μmol L^−1^) induced 465 DEGs, of which 212 were upregulated and 253 were downregulated. GO classification results showed that, as shown in Figure 2b, multiple genes related to oxidation reduction, DNA replication, cell cycle processes, metabolism of xenobiotics by cytochrome, signaling pathways, extracellular matrix, and response to external biotic stimuli were significantly enriched following exposure to La and Gd. In addition, differentially expressed genes were involved in transport, regulation of transcription, and catalytic activity.

**Figure 3:**
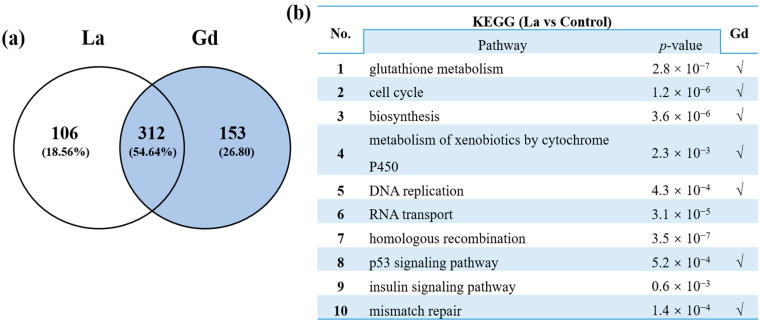
Analysis of the shared differentially expressed genes between La and Gd: (**a**) Venn diagrams showing the differentially expressed genes shared in response to La and Gd exposure; (**b**) top 10 significantly enriched KEGG pathways shared in response to La exposure. √ Indicates the pathways involved in Gd exposure.

KEGG pathway enrichment analysis was performed for the DEGs in each treatment group. As indicated in Figure 3, most of the DEGs in *Danio rerio* were enriched in glutathione metabolism, cell cycle, biosynthesis, metabolism of xenobiotics by cytochrome P450, DNA replication, mismatch repair, homologous recombination, p53 signaling pathway, and insulin signaling pathway. Interestingly, we found that DEGs induced by La and Gd share a considerable number of common enrichment pathways. These results suggest that La and Gd may share similar biological mechanisms of action.

### 3.3. Validation of DEGs Induced by La and Gd

**Figure 4:**
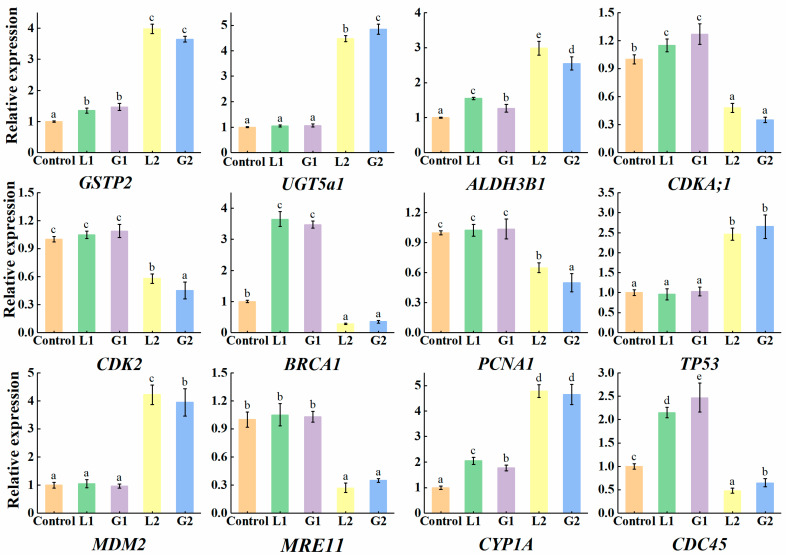
Differential expression in liver tissues of *Danio rerio* exposed to La and Gd: L1, 15μmol L^−1^ La; L2, 30 μmol L^−1^ La; G1, 15μmol L^−1^ Gd; and G2, 30 μmol L^−1^ Gd. Data are shown as mean ± SD by qPCR. The expression of the control was set as 1. Data presented are the average of three replicates. Housekeeping gene *ACT2* was used as an internal control, the same below. Different letters indicate significant differences in gene expression.

qRT-PCR was used to further verify that La and Gd induced the DEGs of *Danio rerio* liver. Twelve DEGs were successfully amplified and compared with those in the transcriptome (Figure 4). In the 30 μmol L^−1^ La treatment group, the relative expression of *GSTP2*, *UGT5a1*, *ALDH3B1*, *CYP1A*, *TP53*, and *MDM2* showed a significant upregulation trend and were 3.98-, 4.45-, 2.95-, 4.73-, 2.51-, and 4.27-times higher than the control, respectively, while the relative expression levels of *CDKA;1*, *CDK2*, *BRCA1*, *PCNA1*, *MRE11*, and *CDC45* were significantly downregulated, showing 0.43-, 0.65-, 0.32-, 0.62-, 0.27-, and 0.49-fold reductions, respectively, compared with the control. The changes in these genes in qPCR and transcriptome sequencing were the same, indicating that the transcriptome sequencing analysis was reliable. Furthermore, compared with La and Gd, there was no significant difference in the change trends of the 12 DEGs, with only a slight difference in the amount of expression (Gd treatment induced a slightly higher number of DEGs than La treatment).

## 4. Discussion

REEs are metals located in the sixth period of the periodic table. According to previous studies, no significant physiological transformation processes associated with REEs have been found in animals; therefore, REEs are not considered essential life elements. Long-term exposure to or consumption of foods containing high levels of REEs may have biological effects. In the present study, the highest enrichment of La and Gd was found in the *Danio rerio* liver, which was 30–60 times higher than that in muscle. Similar results have also been reported in other studies. Tu [31] studied the accumulation of REEs in different organs of carp and found that the bioenrichment in carp was in the following order: liver > gills > bone > muscle. Bustamante [32] found that invertebrates have the highest enrichment of REEs in their digestive glands, followed by the gills, gonads, kidneys, and muscles. Therefore, the liver of *Danio rerio* was used as the target organ in this study to explore the toxic effects of the selected two REEs.

Whether REEs are genotoxic remains controversial. Generally, REEs have a significant hormesis effect: low doses promote biological growth, whereas high doses have a negative effect [33]. The growth-promoting effect of REEs is related to the promotion of cell proliferation, similar to that of calcium (Ca) [34]. At high doses, REEs cause various types of base damage, DNA breakage, mutation, and carcinogenesis [35]. Ca plays an important role in cell growth and proliferation [36,37]. REEs and Ca have the same ion radius; therefore, REEs can act as substitutes for extracellular or intracellular Ca, resulting in the disruption of Ca homeostasis and leading to disorders of Ca-related biological processes [37,38]. From the findings of the present study, La and Gd can be postulated to interfere with the normal operation of physiological activities involved in cytoplasmic Ca, thus interfering with the expression of biomolecules, including cyclin B, insulin growth factor, and cyclin-dependent kinase, further affecting the progression of cell meiosis.

Environmental stress can cause various forms of DNA damage. To maintain the integrity of the genome, organisms can activate the activity of DNA repair complexes through signal cascade reactions, regulate the process of the cell cycle, or start the process of cell death [39]. The expression of cell cycle regulatory genes (such as *BRCA1*, *MRE11*, and *TP53*) are regulated by ataxia telangiectasia mutated (ATM)/ataxia telangiectasia and Rad3 (ATR) related dependent signal transduction [40]. They play an important role in monitoring genomic integrity and DNA damage repair [41]. In this study, when La and Gd concentrations were 15 μmol L^−1^, the expression of *BRCA1* in the liver of *Danio rerio* increased by 3.67 times compared with the control. The increased expression of genes related to the cell cycle and damage repair reduced the damage in the liver of *Danio rerio*. When La and Gd concentrations were 30 μmol L^−1^, the expression of *BRCA1* decreased significantly, which indicated that a high concentration of La induced DNA damage. Similarly, the expression of *MRE11*, which is related to the terminal repair of DNA double-strand breaks (DSB) [42], decreased significantly in the 30 μmol L^−1^ La and Gd treatment groups. These results showed that under high concentrations of REEs treatments, the expression of damage repair-related genes decreased, and the dysfunctional repair system increased DNA damage in the liver of *Danio rerio*. At the same time, the increased expression of *TP53* and *MDM2* indicated that the cells might undergo apoptosis.

Herein, several processes related to exogenous substance metabolism (such as the metabolism of xenobiotics by cytochrome P450 and glutathione metabolism) were significantly enriched following exposure to La and Gd. Several important enzyme-encoding genes involved in the metabolism of exogenous substances, such as GSTP2, UGT5a1, and Aldh3b1, were significantly upregulated following exposure to La and Gd. Glutathione is an indispensable substrate for the catalytic detoxification of glutathione sulfotransferase [43,44]. Acetaldehyde dehydrogenase, the expression product of the Aldh3b1 gene, is also an important enzyme for cellular detoxification, especially for cytotoxicity caused by oxidants [45]. When an organism is under adverse stress or disturbed by foreign substances, the organism initiates relevant mechanisms for the repair or maintenance of homeostasis, and a series of detoxifying metabolic enzymes are activated, and their expression levels are elevated to increase cellular tolerance [46]. In the present study, the observation of enriched detoxification metabolic pathways and the upregulated expression levels of some key metabolic enzyme genes suggest that exposure to these two REEs stimulated defense mechanisms in the zebrafish. The results of GO annotation confirmed the aforementioned postulation, with the response to external biotic stimulus and the extracellular matrix significantly enriched in both the La and Gd treatment groups.

Whether there is a common toxicity mechanism or modes of action among REEs has always been a question. Because they have the same number of electronic layers and outer electronic structure, all REEs possess very similar chemical properties and are, thus, regarded as a group of elements with uniform chemical properties [11,47]. There has been speculation about the homogeneity of REEs; however, there are not enough comparable data to reach a consistent conclusion [21]. Previous acute toxicity experiments showed that there was no significant difference in lethality between the two elements. However, Gd may cause a stronger molecular response to some biological processes and induce more DEGs than La (Figure 4). Similarly, an experiment on sea urchin embryos showed that the levels of reactive oxygen species and malondialdehyde were higher following Ce than La exposure [48]. However, of the two, Ce did not have a stronger effect on most biological processes. In a study of two sea urchin species, La caused damage to embryos, whereas Ce did not [10]. Herein, both La and Gd had similar lethality; however, a comparison of their toxicity patterns needs to be based on the investigated species, toxicological endpoints, and physiological processes.

In conclusion, in this study, there were numerous DEGs and biological pathways co-enriched by La and Gd, while only a few biological processes were affected by one element. The expression of genes related to cell division, exogenous substance metabolism, and some basic physiological activities, such as DNA replication and the cell cycle, differed slightly between the two treatments; however, the complete physiological process was subjected to the same interference under La and Gd stress. Taken together, La and Gd may affect some biological processes with different intensities; nonetheless, there are many similarities in their overall toxicity mechanisms and modes of action. The current data help clarify the potential toxicity and modes of action of REEs, which are not yet fully recognized, thereby contributing to their environmental risk assessment.

## Figures and Tables

**Table 1:** La and Gd uptake and bioconcentration factors (*BCF*) in muscle, gills, and liver tissues of *Danio rerio* exposed to 15 and 30 μmol L^−1^ La and Gd for 28 days, respectively.

Concentration (μmol L^−1^)		Uptake (μg g^−1^, Fresh Weight) (*BCF*)		
		Muscle	Gills	Liver
15	La	0.53 ± 0.14 ^b^ (0.25)	9.36 ± 1.56 ^b^ (4.49)	19.45 ± 3.21 ^b^ (9.33)
	Gd	0.41 ± 0.09 ^a^ (0.17)	8.43 ± 1.22 ^a^ (3.58)	18.21 ± 3.68 ^a^ (7.73)
30	La	1.38 ± 0.26 ^c^ (0.33)	20.35 ± 3.72 ^d^ (4.88)	35.41 ± 8.94 ^c^ (8.49)
	Gd	1.22 ± 0.35 ^c^ (0.26)	15.21 ± 2.54 ^c^ (3.23)	39.85 ± 7.62 ^d^ (8.46)

Mean values ± standard deviation are shown (*n* = 3); different letters next to the values indicate a significant difference (*p* < 0.05) among the different treatment groups, the same below: *BCF*, REEs concentration in muscle, gills, or liver/in solution.

## Data Availability

Data can be made available upon reasonable request.

## Supplementary Materials

The following are available online at https://www.mdpi.com/article/10.3390/toxics10090519/s1, Table S1: Molarity of each ion in the standard water; Table S2: Ammonia concentration (mg/L) measurements during 72-h acute toxicity; Table S3: Primer sequences used in the experiment; Table S4: Analysis of the water quality of the solution during the toxicity test; Table S5: Gene ontology and gene symbol in zebra fish livers induced by La and Gd. References [49,50,51,52] are cited in the supplementary materials.
