# Supplementary material for: Toxic Effects of Two Representative Rare Earth Elements (La and Gd) on Danio rerio Based on Transcriptome Analysis
Source: Toxics. 2022 Aug 31;10(9):519. doi: 10.3390/toxics10090519 (PMC9503537; doi:10.3390/toxics10090519)
Supplement: Supplementary file 1 [file toxics-10-00519-s001.zip › toxics-1844700-supplementary.pdf]

toxics

Supplementary Materials: Toxic Effects of Two Representative Rare Earth Elements (La and Gd) on Danio Rerio Based on Transcriptome Analysis

Shu Kang, Cheng Guo, Chenyang Xue, Chenshu Ma, Huaizhong Mu and Lizong Sun

Table S1. Molarity of each ion in the standard water.

Standard Water [a]	Cations (mmol·L⁻¹)				Anions (mmol·L⁻¹)			Hardness [b]	pH
	Ca^{2+}	Mg^{2+}	Na^{+}	K^{+}	HCO_3^-	Cl^-	SO_4^{2-}		
	2.0	0.5	0.8	0.08	0.8	4.08	0.5	250	7.5–7.8

a: based on the OECD 203 and GB/T13267-91.
b: hardness is calculated by Ca^{2+}-Mg^{2+} content.

Table S2. Ammonia concentration (mg/L) measurements during 72 h acute toxicity.

Exposure Concentration (μmol L⁻¹)	0	24 h	48 h	72 h
**0**	-	0.03±0.005	0.04±0.006	0.02±0.006
**20**	-	0.04±0.004	0.05±0.015	0.05±0.007
**40**	-	0.08±0.012	0.07±0.013	0.08±0.010
**80**	-	0.10±0.021	0.08±0.017	0.06±0.014
**160**	-	0.07±0.016	0.03±0.011	0.01±0.002
**320**	-	0.06±0.014	- (all fish dead)	- (all fish dead)

-: not detected.
Total ammonia was measured according to the Nessler's Reagent Spectrophotometry method. The determination method referred to the previous studies [1–2].

Table S3. Primer sequences used in the experiment.

Genes	Full Name	Primer Sequence (5′–3′)	Product Size (bp)
CDKA;1	Cyclin-dependent kinases A;1	F: CCTGTCAGGACATTTACTCATGAG<br>R: GCTTTTGGCTGATCATCTCAGC	149
CDK2	Cyclin-dependent kinases 2	F: GTAGTAAGGTCTTCTGCAAGGCA<br>R: TGCCATTCCAACATATGTGC	138
**GSTP2**	Glutathione S-transferase protein 2	F: ACCATCCACCGTCATCTC<br>R: GCTCCTGTCGTTATTATTACTG	147
**UGT5a1**	Uridine 5′-diphosphate-glucuronyl transferase a1	F: CTCAAAATCCCACGCTTCTTGTGG<br>R: CACGTCTACTACCTTTGGTTTCCC	180
**ALDH3B1**	aldehyde dehydrogenase 3 family, member B1	F: TGGTGCTGGACATTTCAGTCGG<br>R: CAAGAGCTTGCACTTCCATCATAG	198
PCNA1	Proliferation cell nuclear antigen 1	F: GTGACACAGTTGTGATCTCTG<br>R: ATCACAATTGCATCTTCCGG	223
**CYP1A**	cytochrome P450, family 1, subfamily A, polypeptide 1	F: GCAAACATCAGAGACCCCGA<br>R: TGCAAGCGACCTGATACTCC	167
CDC45	cell division cycle gene 45	F: TAGCCAATGCTCGCCATGAA<br>R: CGTCGGATTGAAATGACGGC	192
TP53	Tumor Protein 53	F: TCTTTGATCTATAGGTTTCCGCCT<br>R: GGACCGGCTTGGTTGTTAGA	184
BRCA1	Breast cancer susceptibility1	F: GATTCGTCGTCTTGCTCGTAG<br>R: CAGTCACCGTCTTCCTCCTC	153
MRE11	MRE11 homolog, double strand break repair nuclease	F: GTGATACACTTCGAGTACTTGTTGC<br>R: CTGACTACTTGAAACTGCACTGG	167
MDM2	murine double minute2	F: GTAACCATGTATTTTGCAATGCGTG	256

		R: GTGACGGATTATTCTGGCTAACG	
ACT2[#]	House-keeping gene AtACT2	F: TCGTGGATTCCAGCAGCTTCC<br>R: CCGATGGGCAAGTCATCACG	105

#: To provide stable housekeeping genes for expression analysis of genes in zebrafish, eight commonly used plant internal reference genes (ACT1, ACT2, U6, aTUB1, eIF2, CYP, ACP, EF1α) [3]. were selected as candidate housekeeping genes based on the transcriptome data. qRT-PCR was used to detect the expression of these candidate genes in the muscle, liver, and gills of zebrafish. Then, the geometric average method (geNorm, average expression stability value, M) was used to analyze the expression stability of the candidate housekeeping genes [4].

Table S4. Analysis of the water quality of the solution during the toxicity test.

Toxicity Test	Day	pH	Temperature (℃)	DO (%ASV)	Weight (g)	Length (cm)	La (μmol L^{-1})	Gd (μmol L^{-1})
72 h	**0**	7.0	26	90	-	-	20; 40; 80; 160; 320	20; 40; 80; 160; 320
	**1**	7.0	25.7	88	-	-	19.5; 38.2; 83.4; 154.3; 311.4	18.7; 41.6; 76.2; 153.7; 325.6
	**2**	6.9	26.1	85	-	-	18.9; 37.7; 75.6; 152.4; 307.8	19.3; 37.6; 78.7; 154.3; 319.4
	**3**	7.0	26.2	79	-	-	18.5; 37.2; 74.3; 151.7; 313.3	18.2; 36.7; 74.3; 149.6; 310.2
96 d	**0**	7.0	26	90	0.18	1.51	15; 30	15; 30
	**1**	7.0	26.1	86	-	-	14.9; 30.2	14.8; 29.4
	**3**	6.8	25.8	90	-	-	15.3; 28.7	15.1; 30.7
	**7**	6.9	26.2	85	-	-	14.2; 28.5	15.7; 28.1
	**10**	7.2	26.1	90	-	-	15.7; 31.2	16.3; 32.1
	**14**	7.0	26	84	-	-	15.1; 28.4	15.2; 30.4
	**17**	7.1	25.8	89	-	-	15.9; 31.6	13.9; 32.3
	**21**	6.8	25.8	83	-	-	13.8; 27.5	13.7; 31.2
	**24**	6.8	26.2	90	-	-	16.3; 32.2	15.8; 30.6
	**28**	7.1	26.1	81	0.25	1.83	13.6; 27.6	14.2; 28.3

Mean values is shown (n = 3); DO: dissolved oxygen; ASV: air saturation value; 0: Setting values; -: not measured.

Table S5. Gene ontology and gene symbol in zebra fish livers induced by La and Gd.

Gene Ontology	*P*-Value	Gene Symbol
oxidation reduction	1.4×10^{-4}	*GSTP2, CYP1A, ZGC:64106, F5, GPX1B, CYP3A65, HSD11B2, CYP3C1L2*
DNA replication	3.5×10^{-6}	*CDC45, MRE11, PCNA1, dbf4b, fen1, chaf1a, mcm2, mcm3, mcm4, prim1, prim2*
cell cycle	2.8×10^{-7}	*BRCA1, CDKA;1, CDK2, WEE1, CDK4, E2F1, CYCB1;1, PCNA2, CCNA1, CCND1, ATM, BRCA1*
metabolism of xenobiotics by cytochrome	3.1×10^{-5}	*UGT5a1, ALDH3B1, PCK1, CPB1, A2M, APOA1, F5, COL10A1, CYP3A65, C1q, C7-1, EGR1*
mismatch repair	3.5×10^{-7}	*TP53, MDM2, RB1, ATM*
transport	5.2×10^{-4}	*APOA1, RBP2A, UCP4, FABP2, UCP2L, FABP1B, FABP6, SLC6A19*
signaling pathway	3.5×10^{-4}	*CCNG2, GADD45AL, ZGC:153369*
regulation of transcription	6×10^{-3}	*EGR1, HER2, IRF2BP1, ZGC:77060*
signal transduction	1.1×10^{-5}	*PDK2, FGA, SI:RP71-57J15.4*

References

1. Liu, Z.; Li, X.; Tai, P.; Sun, L.; Yang, X. Toxicity of ammonia, cadmium, and nitrobenzene to four local fishes in the Liao River, China and the derivation of site-specific water quality criteria. *Ecotoxicol. Environ. Saf.* **2017**, 147, 656–663. https://doi.org/10.1016/j.ecoenv.2017.09.008.
2. Liu, Z.; Tai, P.; Li, X.; Kong, L.; Matthews, T.G.; Lester, R.E.; Mondon, J.A. Deriving site-specific water quality criteria for ammonia from national versus international toxicity data. *Ecotoxicol. Environ. Saf.* **2019**, 171, 665–676. https://doi.org/10.1016/j.ecoenv.2018.12.078.
3. Li, X.; Yang, Y.; Ahmad, S.; Sun, M.; Zhang, Q. Selection of optimal reference genes for qRT-PCR analysis of shoot development and graviresponse in prostrate and erect chrysanthemums. *PLoS ONE* **2019**, 14, e0225241. https://doi.org/10.1371/journal.pone.0225241.

4. Louren, A.P.; Mackert, A.; Cristino, A. Validation of reference genes for gene expression studies in the honey bee, apis mellifera, by quantitative real-time rt-pcr. *Apidologie* **2008**, 39, 372–385. https://doi.org/10.1051/apido:2008015.
